# Impact of dedicated neuro-anesthesia management on clinical outcomes in glioblastoma patients: A single-institution cohort study

**DOI:** 10.1371/journal.pone.0278864

**Published:** 2022-12-13

**Authors:** Jasper Kees Wim Gerritsen, Dimitris Rizopoulos, Joost Willem Schouten, Iain Kristian Haitsma, Ismail Eralp, Markus Klimek, Clemens Maria Franciscus Dirven, Arnaud Jean Pierre Edouard Vincent

**Affiliations:** 1 Erasmus Medical Center Rotterdam, Department of Neurosurgery, Rotterdam, The Netherlands; 2 Erasmus Medical Center Rotterdam, Department of Biostatistics, Rotterdam, The Netherlands; 3 Erasmus Medical Center Rotterdam, Department of Anesthesiology, Rotterdam, The Netherlands; CHOC Children’s Hospital - UC Irvine, UNITED STATES

## Abstract

**Background:**

Glioblastomas are mostly resected under general anesthesia under the supervision of a general anesthesiologist. Currently, it is largely unkown if clinical outcomes of GBM patients can be improved by appointing a neuro-anesthesiologist for their cases. We aimed to evaluate whether the assignment of dedicated neuro-anesthesiologists improves the outcomes of these patients. We also investigated the value of dedicated neuro-oncological surgical teams as an independent variable in both groups.

**Methods:**

A cohort consisting of 401 GBM patients who had undergone resection was retrospectively investigated. Primary outcomes were postoperative neurological complications, fluid balance, length-of-stay and overall survival. Secondary outcomes were blood loss, anesthesia modality, extent of resection, total admission costs, and duration of surgery.

**Results:**

320 versus 81 patients were operated under the anesthesiological supervision of a general anesthesiologist and a dedicated neuro-anesthesiologist, respectively. Dedicated neuro-anesthesiologists yielded significant superior outcomes in 1) postoperative neurological complications (early: p = 0.002, OR = 2.54; late: p = 0.003, OR = 2.24); 2) fluid balance (p<0.0001); 3) length-of-stay (p = 0.0006) and 4) total admission costs (p = 0.0006). In a subanalysis of the GBM resections performed by an oncological neurosurgeon (n = 231), the assignment of a dedicated neuro-anesthesiologist independently improved postoperative neurological complications (early minor: p = 0.0162; early major: p = 0.00780; late minor: p = 0.00250; late major: p = 0.0364). The assignment of a dedicated neuro-oncological team improved extent of resection additionally (p = 0.0416).

**Conclusion:**

GBM resections with anesthesiological supervision of a dedicated neuro-anesthesiologists are associated with improved patient outcomes. Prospective evidence is needed to further investigate the usefulness of the dedicated neuro-anesthesiologist in different settings.

## Introduction

### Background

Glioblastomas (GBM) are malignant brain tumors with an annual incidence of six per 100,000. The standard treatment consists of surgery with adjuvant chemoradiotherapy. Due to GBMs infiltrative nature, they generally have a relatively poor sensitivity to adjuvant therapy and are invariably lethal. The median overall survival of GBM is approximately 15 months [1–3]. Due to the limited prognosis of these patients, considerable efforts should be aimed at preserving their quality of life (QoL) by maximizing the extent of resection while minimizing postoperative neurological complications [4, 5].

Recently, the contribution of the anesthesiologist to patient outcomes was a topic of discussion since a broad variability has been found [6]. In most countries, GBM resections are mostly performed under the supervision of a general anesthesiologist, rather than a dedicated neuro-anesthesiologist. These dedicated specialists are scheduled predominantly for more complex cases (e.g. patients with tumors that are difficult to approach or patients with notable comorbidities).

As to date, there is no research available regarding the clinical outcomes of glioma patients operated under the supervision of general anesthesiologists versus dedicated neuro-anesthesiologists. Hence, we strive to lay the framework for further research by evaluating the outcomes of these two groups of GBM patients in a large retrospective cohort of patients from our institution. We aim to determine whether the assignment of a dedicated neuro-anesthesiologist to GBM resections results in improved outcomes of these patients.

## Patients and methods

### Participants

The cohort consisted of 438 supratentorial GBM patients who had undergone tumor resection at our instition between January 2008 and July 2017. 401 patients were operated under general anesthesia (without surgical adjuncts), 37 patients were operated with intraoperative stimulation mapping using awake craniotomy (AC; no asleep mapping techniques were performed). We decided to exclude AC patients from this study, since 1) AC is used in GBM surgery, for a major part, to minimize postoperative neurological complications, which would introduce a confounder and 2) all the AC patients were operated under the supervision of a neuro-anesthesiologist, which would introduce a bias. In all cases, neuronavigation was used. Cases performed with surgical adjuncts such as 5-ALA (5-aminolevulinic acid, Gliolan®, Specialised Therapeutics Australia) and intraoperative ultrasound were included. No cases with intraoperative MRI were included since this modality is not available at our institution.

Eligibility criteria were: 1) isolated GBM without evidence of multicentric or multifocal enhancement; 2) pathological diagnosis of glioblastoma multiforme (WHO Grade IV); 3) supratentorial lesion; 4) preoperative KPS ≥60 and 5) planned/scheduled surgery.

### Setting

All patients included in this study were operated under GA. The anesthesiologist chose one of three options for anesthesia maintenance: 1) a volatile anesthetic such as isoflurane or sevoflurane balanced with intravenous opioids; 2) TIVA (Total IntraVenous Anesthesia) with propofol and intravenous opioids; 3) a combination of a volatile anesthetic and propofol with intravenous opioids. The procedure remained constant throughout the cohort. Patients were intubated after single-bolus muscle relaxation with rocuronium or cis-atracurium and mechanically ventilated throughout the procedure. Arterial blood pressure was measured invasively via the radial artery, and all patients received a urinary catheter. Mannitol 15% was given preoperatively and/or peroperatively on discretion by the anesthesiologist or as requested by the neurosurgeon in case of relevant edema. Local anesthesia of the surgical field was performed with 10 ml lidocaine 1% and adrenaline 1:200.000. After surgical incision, craniotomy and opening of the dura, the tumor was removed using BrainLab© neuronavigation. No mapping techniques for speech or motor function using cortical or subcortical electrostimulation were used to resect the tumor. After the operation, all patients were brought to the post-anesthesia-high-care-unit (PACU), where they spent the first 24 hours postoperatively. On this high-dependency, the nurses work under supervision of a dedicated staff anesthesiologist (not necessarily the one who provided the intraoperative anesthesiological care) and follow standard protocols for all patients who have undergone brain tumor resections.

Procedures were allocated to dedicated neuro-anesthesiologists either in a random manner (the neuro-anesthesiologist was fortuitously the scheduled anesthesiologist for the respective case, estimated at 80–85% of cases) or specifically pre-planned (the procedure was deemed by the neurosurgeon as rather difficult due to the nature of the procudure or serious comorbidities and the planning was made depending on the availability of the dedicated anesthesiologist (estimated at 15–20% of cases)). Patients were included in the general anesthesiologist group or the dedicated neuro-anesthesiologist group based on the anesthesiologist responsible for the procedure. Dedicated neuro-anesthesiologists in this study were defined as anesthesiologists who perform >75% of their clinical activities in the perioperative care for neurosurgical patients and who have followed an accredited (inter)national dedicated training or -fellowship in which they have been exposed to an expansive neurosurgical caseload both quantitatively as qualitatively as well as having developed a research niche in the subspecialty. During the study period, there were two dedicated neuro-anesthesiologists (I.E. and M.K.), with more than 10 years of training as a staff member after their dedicated training. In general, on all days of the week one of them is available, whilst the neurosurgeons mostly have fixed days of the week on which they operate. This might contribute to fixed combinations between neurosurgeons and dedicated neuro-anesthesiologists. General anesthesiologists, on the other hand, were defined as anesthesiologists who are ‘allround’ in their daily clinical activities and do not perform the majority of those (activities) in the care for neurosurgical patients, nor have they followed dedicated training or -fellowships or performed research in this area. They rotate every day between different subspecialities. On average, the neurosurgical caseload for a dedicated neuro-anesthesiologist in our department is around 200 cases per year, of which 120–140 brain tumor operations. For general anesthesiologists, the average neurosurgical caseload is 40–50 cases per year (mostly neurosurgical emergencies) of which <20 brain tumor operations. We also looked at dedicated oncological neurosurgeons as a variable in this study. This group is defined as neurosurgeons whose operative load consists of oncological surgery for >80%. At our institution, there are four dedicated oncological neurosurgeons who have all more than 15 years of training as a staff member after their oncological neurosurgery fellowship.

### Variables

Patient characteristics were retrospectively collected the hospital records and screened for presenting symptoms, preoperative patient functioning and fitness (KPS, ASA), neuroimaging findings, neurological functioning, intraoperative variables and adjuvant treatment. Ethical approval had been obtained and the requirement for written informed consent was waived by the IRB (METC Erasmus MC, Rotterdam, The Netherlands, MEC-2020-0811). The study has been conducted in compliance with the principles of the Declaration of Helsinki (2013) and the General Data Protection Regulation (GDPR) (2018). Preoperative KPS is determined routinely by the clinician at the time of evaluation. Total admission costs were calculated as: (days in PACU*PACU cost/day) + (days in neurosurgery ward*ward cost/day). The recorded MRI characteristics included the lesion’s size, specific lobe involvement, multifocality, and extent of resection The lesion’s size was calculated preoperatively and postoperatively manually calculated based on T1 with contrast MR images using the frequently used method described by (among others) Shah and colleagues [7], which was approved by the neuroradiology department. Extent of resection (EOR) was calculated as (preoperative tumor volume—postoperative tumor volume)/preoperative tumor volume and was calculated based on the contrast-enhanced tumor on MRI T1 + Gd contrast images. Postoperative neurological complications were classified in four categories: early minor-, early major-, late minor-, and late major complications. Classification of postoperative neurological complications was used as described in the meta-analysis of colleagues de Witt Hamer and colleagues [8]. Major complications included hemiparesis, monoparesis MRC grade 1–3, aphasia or severe dysphasia, hemianopsia, visual field complications and vegetative state. Minor complications included monoparesis MRC grade 4, N. VII palsy, dysnomia, somatosensory syndrome, parietal syndrome or isolated cranial nerve deficit. The distinction between early- and a late neurological complication was 3 months postoperatively, which is the usual cutoff point for permanency of postoperative neurological complications. Postoperative neurological complications were assessed by retrospectively evaluating the electronic patient records.

Primary outcomes were 1) postoperative neurological complication rate, 2) fluid balance (ml deviating from zero; total IV fluid volume minus all fluids lost), 3) length-of-stay (LOS) and 4) overall survival (months). Secondary outcomes were blood loss (ml), anesthesia modality, extent of resection (%), total admission costs (EUR) and OR duration (min).

### Statistical methods

Statistical analyses were executed in collaboration with a senior statistician from the Department of Biostatistics of our own institution. Differences between the patients receiving anesthesia from a general anesthesiologist or a dedicated neuro-anesthesiologist for the primary and secondary outcomes were tested with univariate analyses. For statistically significant outcomes a multivariate analysis was performed in addition to the univariate analysis to minimize the risk for confounders and selection bias. Subgroup analyses were done for the assignment of a neuro-anesthesiologist and a oncological neurosurgeon to GBM cases. Analyses of differences between two groups in baseline characteristics for continuous variables were done using the two-tailed *t* test for independent samples. For categorical variables, the chi-square (χ^2^) test was used. Analysis of the data set for outcomes based on continuous variables was done using the Kruskal-Wallis test, whereas for categorical variables the Fisher-exact test was used. Analysis of overall survival was done with the log-rank test. Significant outcomes in the univariate analysis were further analysed using a multivariate analysis using standard logistic regression. The multivariate analysis consisted of well known predictive factors in glioblastoma patients: 1) age; 2) gender; 3) preoperative KPS; 4) tumor location and 5) preoperative tumor volume. For the comparison of multiple subgroups regarding the composition of the performing team, the one-way analysis of variance (ANOVA) test was used for continuous variables and the chi-square (χ^2^) test for categorical variables. The significance level for all tests was set to 5%.

## Results

### Baseline characteristics

After excluding all awake cases (n = 37), a total of 401 patients were included in the cohort. All of the included patients had undergone GBM resection under general anesthesia. 320 patients were operated on with anesthesiological supervision by a general anesthesiologist (GA-group), whereas in 81 patients a dedicated neuro-anesthesiologist provided anesthesia care (NA-group). Baseline patient characteristics for both groups are shown in Table 1. No significant differences in baseline characteristics between groups were observed for demographics, adjuvant treatment, preoperative tumor volume, tumor location, proportion of eloquent areas, preoperative patient performance (KPS and ASA scores), proportion of resections done by a dedicated oncological neurosurgeon or the use of 5-ALA fluorescence. A significant difference was found for the use of intraoperative ultrasound, which was used more frequently in the neuro-anesthesia group (14.8% vs. 5.3%; p = 0.0102).

**Table 1:** Baseline characteristics.

Characteristic	Neuro-anesthesiologist	General anesthesiologist	*P* value
Total *n* patients	81	320	
Demographics			
Mean age (yrs)	59.2	58.6	*p* = 0.699
Range	19–80	18–80	
Gender			
Male (%)	53 (65.4)	186 (58.1)	*p* = 0.231
Female (%)	28 (34.6)	134 (41.9)	
Adjuvant treatment (%)			
Chemoradiation	55 (67.9)	199 (62.2)	*p* = 0.373
Chemotherapy	3 (3.7)	4 (1.3)	
Radiation	17 (21.0)	79 (24.7)	
None	5 (6.2)	34 (10.7)	
Unknown	1 (1.2)	4 (1.3)	
Tumor volume			
Mean tumor volume in mm3 (SD)	63663 (49111)	54064 (44049)	*p* = 0.0878
Tumor location–lobe (%)			
Frontal	22 (27.2)	138 (30.2)	*p* = 0.632
Parietal	17 (21.0)	91 (19.9)	
Temporal	38 (46.9)	190 (41.6)	
Occipital	4 (4.9)	38 (8.3)	
Tumor location–hemisphere (%)			
Right	41 (50.6)	214 (46.8)	*p* = 0.529
Left	40 (49.4)	243 (53.2)	
Tumor location–eloquent areas (%)	53 (65.4)	178 (55.6)	*p* = 0.138
Patient performance			
Median preoperative KPS (range)	90 (60–100)	90 (60–100)	*p* > 0.05
Median ASA score (range)	II (I-III)	II (I-III)	*p* > 0.05
Dedicated oncological neurosurgeon (%)	49 (60.5%)	182 (56.9%)	*p* = 0.559
Surgical adjuncts			
5-ALA fluorescence	6 (7.4%)	9 (2.8%)	*p* = 0.0891
Intraoperative ultrasound	12 (14.8%)	17 (5.3%)	*p* = 0.0102*

### Primary outcomes

First, patients in the NA-group experienced fewer postoperative neurological complications (Tables 2 and 3). Early major complications were less frequent in the NA-group than in the GA-group (13.6% and 28.8%; p = 0.0045, overall multivariate analysis: p = 0.002, OR = 2.54, Table 2). This was also the case for late minor- and late major complications: 12.3% late minor complications in the NA-group versus 30.9% in the GA group (p = 0.0008); 16.0% late major complications in the NA-group versus 26.9% in the GA group, (p = 0.044), overall multivariate analysis: p = 0.003, OR = 2.24, Table 2). However, early minor complications did not differ between groups (p = 0.227).

**Table 2:** Primary and secondary outcomes.

					Univariate analysis	Multivariate analysis		
Variable	Anesthesiologist	*n* total	Neuro-anesthesiologist (SD)	General anesthesiologist (SD)	*P* value	*P* value	SE	*t* value
Mean fluid balance (ml)	neuro-anesthesiologist	77	-232.1 (11.2)	538.3 (11.2)	*p* < 0.0001	*p* < 0.001*	112.0	6.41
	general anesthesiologist	309						
	all	386						
Mean LOS (days)	neuro-anesthesiologist	81	6.3 (3.8)	7.8 (4.8)38.90	*p* = 0.0006	*p* = 0.011*	0.57	2.54
	general anesthesiologist	320						
	all	401						
Mean total admission costs (EUR)	neuro-anesthesiologist	81	4709.0 (1927.7)	5471.3 (2432.6)	*p* = 0.0006	*p* = 0.011*	289.28	2.54
	general anesthesiologist	320						
	all	401						
Total amount of propofol (mg)	neuro-anesthesiologist	72	1844.0 (993.1)	2151.0 (922.5)	*p* = 0.005	*p* = 0.028*	152.68	2.21
	general anesthesiologist	81						
	all	153						
Mean extent of resection (%)	neuro-anesthesiologist	81	74.1 (24.6)	68.4 (27.0)	*p* = 0.15			
	general anesthesiologist	320						
	all	401						
Mean OR duration (min)	neuro-anesthesiologist	80	219.6 (64.3)	233.1 (71.8)	*p* = 0.31			
	general anesthesiologist	320						
	all	400						
Mean amount of blood loss (ml)	neuro-anesthesiologist	77	394.4 (337.2)	445.7 (733.5)	*p* = 0.78			
	general anesthesiologist	309						
	all	386						
Median overall survival (months)	neuro-anesthesiologist	80	13 (95% CI: 11–18)	13 (95% CI: 12–16)	*p* = 0.17			
	general anesthesiologist	320						
	all	400						

**Table 3:** Summary statistics of anesthesia modality and neurological complication profile specified by anesthesiologist.

					Univariate analysis	Multivariate analysis		
Factor		*n* total	Neuro-anesthesiologist	General anesthesiologist		*P* value, Odds ratio	SE	*z* value
Anesthesia modality	anesth.vap/comb	82	34	48				
	TIVA	319	47	272				
	all	401	81	320	*p* < 0.0001*	*p* < 0.0001*	0.280	4.93
						OR = 3.97		
Early complications	No	225	59	166				
	Yes	176	22	154				
	Minor	73	11	62	*p* = 0.227			
	Major	103	11	92	*p* = 0.0045*			
	All	401	81	320	*p* < 0.0003*	*p* = 0.002*	0.300	3.11
						OR = 2.54		
Late complications	No	193	58	135				
	Yes	208	23	185				
	Minor	109	10	99	*p* = 0.0008*			
	Major	99	13	86	*p* = 0.044*			
	all	401	81	320	*p* < 0.0001*	*p* = 0.003*	0.276	2.92
						OR = 2.24		

**Fig 1:**
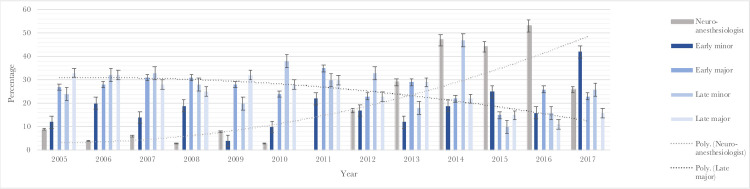
Postoperative neurological complications specified by severity and year (separately uploaded).

Neurological complications were specified by severity, timing and type of anesthesiologist (Table 4). Notably, late postoperative complications were in the GA group more frequently permanent (53.0%) than in the NA group (30.4%) (p = 0.0414). Moreover, neurological complications were specified by year (Fig 1), which also shows that the fraction of resections done under the supervision of a dedicated neuro-anesthesiologist steadily rose over the years. From 2005–2011, less than 10% of GBM resections each year were led by a dedicated neuro-anesthesiologist. From 2012–2014 this number increased from 17 to 47% and remained constant thereafter. Over the years, the incidence of early minor-, early major- and late minor complications remained stable. However, the incidence of late major complications decreased from around 30% to 10–15%.

**Table 4:** Postoperative neurological complications specified by severity, timing and type of anesthesiologist.

	Neuro-anesthesiologist Early (*%*)	Neuro- anesthesiologist Late (*%*)	General anesthesiologist Early (*%*)	General anesthesiologist Late *(%*)
**Minor complications** Monoparesis grade 4 N. VII palsy Dysnomia Somatosensory syndrome Parietal syndrome Cranial nerve deficit	**11 (13.6%)** 4 (4.9%) 0 (0.0%) 2 (2.5%) 3 (3.7%) 2 (2.5%) 0 (0.0%)	*p* = 0.227 **10 (12.3%)** 2 (2.5%) 1 (1.2%) 2 (2.5%) 2 (2.5%) 3 (3.7%) 0 (0.0%)	**62 (19.4%)** 10 (3.1%) 14 (4.4%) 9 (2.8%) 15 (4.7%) 10 (3.1%) 4 (1.3%)	*p* = 0.0045* 99 (30.9%) 11 (3.4%) 26 (8.1%) 20 (6.3%) 22 (6.9%) 13 (4.1%) 7 (2.2%)
**Major complications** Hemiparesis Monoparesis grade 1–3 Aphasia Dysphasia Hemianopsia Visual field deficit unspecified Vegetative/deceased	**11 (13.6%)** 1 (1.2%) 3 (3.7%) 4 (4.9%) 2 (2.5%) 1 (1.2%) 0 (0.0%) 0 (0.0%) 0 (0.0%)	*p* = 0.0008* 13 (16.0%) 2 (2.5%) 0 (0.0%) 4(4.9%) 0(0.0%) 1(1.2%) 3 (3.7%) 3 (3.7%) 0 (0.0%)	**92 (28.8%)** 22 (6.9%) 7 (2.2%) 28 (8.8%) 6 (1.8%) 15 (4.7%) 13 (4.1%) 1 (0.3%) 0 (0.0%)	*p* = 0.044* 86 (26.9%) 18 (5.6%) 4 (1.3%) 25 (7.8%) 6 (1.9%) 7 (2.2%) 20 (6.3%) 5 (1.6%) 1 (0.3%)
**Permanent** **New** **Total**		**7 (30.4%)** **16 (69.6%)** **23 (100%)**		**98 (53.0%)** *p* = 0.0414* 87 (47.0%) *p* = 0.0276* 185 (100%)

Second, fluid management was significantly controlled more strictly (defined as a balance close to 0 or slightly negative) in operations when a dedicated neuro-anesthesiologist was responsible: the mean fluid balance was -232.1 ml in those operations, whereas this was +538.3 ml when a general anesthesiologist was responsible (p<0.0001, multivariate analysis: p<0.001) (Table 2).

Third, after operations for which a dedicated neuro-anesthesiologist was appointed, patients were discharged from the hospital sooner: they stayed on average 6.3 days in the hospital versus on average 7.8 days after an operation led by a general anesthesiologist (LOS) (p = 0.0006, multivariate analysis: p = 0.011) (Table 2).

Fourth, postoperative overall survival did not differ between groups: both groups had a median OS of 13 months (95% CI: 11–18 months in the neuro-anesthesiology group vs. 12–16 months in the general anesthesiology group).

### Secondary outcomes

General anesthesiologists use different anesthesia maintenance techniques than neuro-anesthesiologists: they used TIVA or a volatile anesthetic, whereas neuro-anesthesiologists used a combination of a volatile anesthetic with TIVA significantly more frequently (p<0.0001). The total admission costs per patient were significantly lower in the neuro-anesthesiology group: 4709.0 EUR (SD = 1927.7 EUR) compared to 5471.3 EUR (SD = 2432.6 EUR) (p = 0.0006, multivariate analysis: p = 0.011) (Table 2). No statistically significant differences were observed between the neuro-anesthesiology and general anesthesiology groups for 1) blood loss (393.4 ml versus 445.7 ml, p = 0.78); 2) mean OR duration (219.6 ±64.3 min versus 233.1 ±71.8 min, p = 0.31) and 3) extent of resection (on average 74.1% versus 68.4%, p = 0.15).

### Subgroup analysis

To evaluate the effect of the assignment of a neuro-anesthesiologist or a oncological neurosurgeon to GBM resections, we performed a subgroup analysis. Table 5 shows the baseline characteristics and surgical outcomes of all four subgroups. Subgroup 1 (n = 49) consists of the GBM resections performed by dedicated neuro-anesthesiologist with an oncological neurosurgeon. Subgroup 2 (n = 182) consists of the GBM resections performed by a general anesthesiologist with an oncological neurosurgeon. Subgroup 3 (n = 31) consists of the GBM resections performed by a neuro-anesthesiologist with a non-oncological neurosurgeon. Subgroup 4 (n = 139) consists of the GBM resections performed by a general anesthesiologist with a non-oncological neurosurgeon. No significant differences in baseline characteristics were found for demographics, preoperative tumor volume, tumor location, proportion of resections in or near eloquent areas, preoperative patient functioning (KPS and ASA scores) and the use of 5-ALA fluorescence. Significant differences were found for adjuvant treatment (for 4 patients their adjuvant treatment was unknown in subgroup 3, in comparison with 1, 1 and 3 patients in subgroups 1, 2 and 4;, p<0.001), lobe (different distribution of parietal and temporal lobes in subgroups, p<0.001), and use of intraoperative ultrasound (significantly more often in the subgroups with a neuro-anesthesiologist, p<0.001). The subgroup analysis illustrates the primary and secondary outcomes between selected subgroups, based on the appointed neurosurgeon and anesthesiologist for the case. When evaluating the GBM resections performed by an oncological neurosurgeon (subgroups 1 and 2, n = 231), the addition of a dedicated neuro-anesthesiologist to a GBM resection improved postoperative neurological complications (p = 0.0162 for early minor complications; p = 0.00780 for early major complications; p = 0.00250 for late minor complications and p = 0.0364 for late major complications), EOR (p = 0.0416) and amount of propofol administered (p = 0.0386) significantly. In contrast, a subanalysis of the GBM resections performed by a dedicated neuro-anesthesiologist (subgroups 1 and 3, n = 81) showed that the addition of an oncological neurosurgeon had no significant effect on these outcomes. Notably, the mean extent of resection and rate of postoperative complications were not significantly higher among patients in the NA-groups (subgroups 1 and 3) who were operated with the use of intraoperative ultrasound (ioUS). The mean EOR in subgroup 1 was 81.3% (SD = 18.62) for patients operated with ioUS vs. 76.4% (SD = 21.22) for patients operated without ioUS, p = 0.741. In subgroup 3, the mean EOR was 62.5% (SD = 37.91) for patients operated with ioUS vs. 70.1% (SD = 28.79) for patients operated without ioUS, p = 0.757). For patients operated with ioUS in subgroup 1, the rate of early minor and early major postoperative complications was 11.1% (versus 12.5% without ioUS, p = 0.904), whereas the rates of late minor and late major postoperative complications were 0% and 11.1%, respectively (versus 10.2% (p = 0.317) and 15.0% (p = 0.764) without ioUS). For patients operated with ioUS in subgroup 3, the rates of early minor and early major postoperative complications were 25.0% and 50.0%, respectively (versus 14.8% (p = 0.603) and 11.1% (p = 0.0488) without ioUS), whereas the rate of late minor and late major postoperative complications was 0% (versus 18.5% (p = 0.347) and 22.2% (p = 0.294) without ioUS).

**Table 5:** Baseline characteristics and surgical outcomes for selected subgroups.

**Factor**	**Subgroup 1** Neuro-anesthesiologist + Oncological neurosurgeon	**Subgroup 2** General anesthesiologist + Oncological neurosurgeon	**Subgroup 3** Neuro-anesthesiologist + Non-onco neurosurgeon	**Subgroup 4** General anesthesiologist + Non-onco neurosurgeon	***P* value**
Total *n* patients	49	182	31	139	
Demographics Mean age (yrs) Gender Male (%) Female (%)	59.3 32 (65.3%) 17 (34.7%)	58.4 111 (61.0%) 71 (39.0%)	58.5 21 (67.7%) 10 (32.2%)	58.8 75 (54.0%) 64 (46.0%)	*p* = 0.969 *p* = 0.321
Adjuvant treatment (%) Chemoradiation Chemotherapy Radiation None Unknown	36 (73.5%) 1 (2.04%) 10 (20.4%) 1 (2.04%) 1 (2.04%)	113 (62.1%) 2 (1.10%) 49 (26.9%) 17 (9.34%) 1 (0.549%)	19 (61.3%) 2 (6.45%) 7 (22.6%) 3 (9.68%) 4 (12.9%)	86 (61.9%) 2 (1.44%) 30 (21.6%) 17 (12.2%) 3 (2.16%)	*p* = 0.0101* p = 0.480 *p* = 0.208 *p* = 0.641 *p* = 0.222 *p* = <0.001*
Tumor volume Mean tumor volume in mm3 (SD)	64244 (53909)	58024 (50065)	62774 (41510)	48842 (34041)	*p* = 0.103
Tumor location–lobe (%) Frontal Parietal Temporal Occipital	14 (28.6%) 15 (30.6%) 19 (38.8%) 1 (2.04%)	62 (34.1%) 36 (19.8%) 70 (38.5%) 14 (7.69%)	8 (25.8%) 2 (6.45%) 19 (61.3%) 3 (9.68%)	62 (44.6%) 37 (26.6%) 24 (17.3%) 15 (10.8%)	*p* = <0.001* p = 0.0638 *p* = 0.0356* p = <0.001* p = 0.278
Tumor location–hemisphere (%) Right Left	27 (55.1%) 22 (44.9%)	88 (48.4%) 94 (51.6%)	23 (74.2%) 8 (25.8%)	70 (50.4%) 69 (49.6%)	*p* = 0.0559
Tumor location–eloquent areas (%)	32 (65.3%)	114 (62.6%)	20 (64.5%)	69 (49.6%)	*p* = 0.0664
Patient performance Median preoperative KPS (range) Median ASA score (range)	90 (60–100) II (I-III)	80 (60–100) II (I-III)	90 (70–100) II (I-III)	80 (60–100) II (I-III)	*p* = 0.145 *p* = 0.376
Surgical adjuncts 5-ALA fluorescence Intraoperative ultrasound	4 (8.2%) 9 (18.4%)	15 (8.2%) 8 (4.4%)	1 (3.2%) 4 (12.9%)	3 (2.2%) 5 (3.6%)	*p* = 0.098 *p* = <0.001*
**Factor**	**Subgroup 1** Neuro-anesthesiologist + Dedicated surgeon	**Subgroup 2** General anesthesiologist + Dedicated surgeon	**Subgroup 3** Neuro-anesthesiologist + Non-dedicated surgeon	**Subgroup 4** General anesthesiologist + Non-dedicated surgeon	***P* value**
Postoperative neurological complications Early minor (%) Early major (%) Late minor (%) Late major (%)	6 (12.2%) 6 (12.2%) 5 (10.2%) 7 (14.3%)	*p* = 0.0162* 40 (22.0%) *p* = 0.00780* 57 (31.3%) *p* = 0.00250* 58 (31.9%) *p* = 0.0364* 53 (29.1%)	*p* = 0.6285 *p* = 0.6235 5 (16.1%) *p* = 0.0440 5 (16.1%) *p* = 0.5572 5 (16.1%) 6 (19.3%)	21 (15.0%) 35 (25.2%) 41 (29.5%) 33 (23.7%)	*p* = 0.195 *p* = 0.0272* p = 0.00955* p = 0.150
Mean EOR (SD)	77.4 (20.7)	*p* = 0.0416* 68.9 (26.8)	*p* = 0.137 69.0 (29.3)	67.7 (27.5)	*p* = 0.174
Mean fluid balance [ml] (SD)	-224.9 (913.2)	*p* = <0.001 415 (834.0)	*p* = 0.938 -242.2 (1028.2)	705.95 (881.8)	*p* < 0.0001*
Mean LOS [days] (SD)	6.5 (4.5)	*p* = 0.0656* 8.0 (5.1)	*p* = 0.550 6.0 (2.3)	7.5 (4.4)	*p* = 0.0527
Mean total admission costs [EUR] (SD)	4461.5 (2566.4)	*p* = 0.0983* 5194.6 (2789.5)	*p* = 0.886 4390.7 (1360.5)	5145.5 (2363.4)	*p* = 0.137
Total amount of propofol [mg] (SD)	1856.2 (1147.0)	*p* = 0.0386* 2192.3 (963.7)	*p* = 0.903 1827.0 (744.3)	2058.6 (833.8)	*p* = 0.241

## Discussion

### Key results

We investigated the added value of a dedicated neuro-anesthesiologist for GBM resections in a cohort of more than 400 patients, which makes it the most extensive and comprehensive study about this subject to date. We found that patients undergoing resection for a single supratentorial glioblastoma under the anesthesiological supervision of a dedicated neuro-anesthesiologist 1) experienced, on average, fewer postoperative neurological complications 2) had their fluid balance controlled more strictly and 3) had a shorter postoperative length of stay which directly resulted in lower total admission costs of the operation.

From 2008 to 2017, an increasing number of GBM resections was led by a neuro-anesthesiologist. Simultaneously, the incidence of early minor, early major and late minor complications remained virtually stable during these years. However, the incidence of late major complications decreased dramatically, from more than 33% in 2005 to 10–15% in the last few years. This might suggest that the implementation of dedicated neuro-anesthesia in GBM resections may be one of the factors that is associated with the decreasing incidence of these neurological complications. Other factors that may contribute to this trend include the improvement in surgical protocols (e.g. increasing use of surgical adjuncts such as 5-ALA and intraoperative ultrasound) and anesthesiological protocols. Our subanalyses suggest that appointing a dedicated neuro-anesthesiologist to GBM cases, irrespective of the expertise of the neurosurgeon (oncological versus non-oncological), provides an added benefit with regard to perioperative and postoperative outcomes,.

### Interpretation

Modern medicine is increasingly developing from specialization towards superspecialization due to the rapid expansion of specialized knowledge in various medical specialties. This holds true for the whole spectrum of medical professionals and is not only limited to adult anesthesiological care. Despite this revolution of specialty and superspecialty in many specialties–including neuroscience and neurosurgery–anesthesiologists are not superspecializing at the same pace [9, 10].

Recently, neurosurgical patients, in particular, have a higher risk of negative outcomes than patients from other disciplines in cases of handovers of the anesthesia care [11]. Anesthesiologists should take note of the recently published neurosurgeons’ wish of “dear anesthesiologist, please don’t abandon us” [12] and in response, provide a continuum of care to every patient, but especially to the GBM patient to optimize their still quite poor outcomes. In 2014, Dr. Ghaly described in his work (published at the SNACC meeting in San Francisco) ‘fifteen reasons that ask for immediate neuroanesthesia commitment and growth in neuroscience’ and the usefulness of a similarly dedicated neuro-care team has already been demonstrated in various studies [13–16]. The main argument for the necessity of these neuro-teams is that the care of the critically ill neurologic patient requires specific training.

We tested different hypotheses to explain why GBM patients who had undergone a resection under the supervision of a neuro-anesthesiologist experienced fewer postoperative complications than patients in the general anesthesiologist group. Generally, GBM surgery means balancing between maximizing the extent of resection while preventing neurological morbidity as much as possible. Therefore, the lower incidence of *early* postoperative complications in the NA-group could have been explained by a safer resection (mapping techniques, for example), or a less extensive resection. Likewise, the lower incidence of *late* postoperative complications in the NA-group could have been explained by a higher extent of resection, potentially resulting in a longer progression-free survival. Since a) the mean extent of resection was not significantly different between the NA-group and GA-group, and b) all patients had been operated without neurophysiological mapping techniques, none of these possible explanations proved to be viable at first.

In order to evaluate the NA-group and GA-group in further detail, we divided each group in two subgroups based on the appointed surgeon (oncological neurosurgeon versus non-oncological neurosurgeon), which resulted in a total of four patient subgroups. We found that the addition of a neuro-anesthesiologist to a GBM resection, irrespective of the surgeon, led to two results that were most notable: 1) fewer postoperative complications, 2) a higher prevalence of ioUS use (5-ALA use was comparable for all subgroups). Moreover, the combination of a neuro-anesthesiologist with a dedicated oncological surgeon (subgroup 1) led to a higher extent of resection. These results indicate that the assignment of a neuro-anesthesiologist certainly helps in achieving a maximum safe resection and that a dedicated neuro-oncological team yields optimal results.

To analyze whether ioUS could explain the lower incidence of postoperative complications in the NA-group, we further evaluated all patients operated with the use of ioUS in both NA-subgroups. We found that mean extent of resection and rate of postoperative complications did not differ between patients operated with or without ioUS in both NA-subgroups (with the exception of late minor complications in subgroup 3 with a p-value of 0.0488 which has to be interpreted with caution regarding the low *n* of ioUS patients in that subgroup). We therefore deem it unlikely that the lower incidence of postoperative complications in the NA-group is caused by the higher prevalence of ioUS use in that group.

We reviewed the anesthesia regimen and found no relevant changes in the protocols during the study period. For example, drugs used, temperature management, and use of neuromonitoring have not undergone significant changes during this period. Also, the use of neuronavigation by the neurosurgeons was used as part of standard-of-care throughout the whole study period.

Based on our dataset, a few potential explanations exist for the fact that in our cohort resections with a neuro-anesthesiologist proved to be safer and more extensive.

First, the lower incidence of late complications in the NA-group may be the result of the higher extent of resection in subgroup 1 (neuro-anesthesiologist with dedicated oncological surgeon), which leads to an increased progression-free survival and consequently, fewer late neurological complications. A second explanation might be the psychological part of appointing a neuro-anesthesiologist to glioblastoma cases: working with an experienced neuro-anesthesiologist makes the surgeon more relaxed and focused, which consequently may have a notable impact on the surgeon’s performance and outcomes. Multiple studies have pointed to the importance of interactive dynamics between surgical team members as key factors for surgical performance and patient outcome [17–19]. Future studies might focus on assessing stress among neurosurgeons (e.g. salivary cortisol) when working with known and less known teams to evaluate this hypothesis further.

A third viable explanation might be the fact that the intravascular volume in NA-led cases proved to be more strictly controlled. Our neuro-anesthesiologists prefer a slightly negative fluid balance (with adequate systematic hemodynamics), which could contribute to less brain edema and subsequent swelling of the brain, which in its turn leads to increased intraoperative field-of-vision and intracranial maneuverability for the surgeon.

While all patients were operated electively and were fasted overnight, their intravascular volume should be normal [20]. Therefore, we hypothesize that the difference of about 750 ml between the groups with an average total amount of fluids given of around 2000 ml might be clinically relevant. Whilst postoperative care is standardized on the PACU, the hemodynamic target-corridors are handed-over by the anesthesiologist involved intraoperatively. We cannot track-back whether there have been differences in these orders (e.g.: maintain MAP between 70–100 mm Hg postoperatively) between the groups, so we cannot exclude the possibility that these potential postoperative differences might have been contributed to differences in outcome.

This results in increased intraoperative safety and decreased postoperative morbidity and could explain the lower incidence of the early postoperative complications predominantly in the NA-group.

Fourth, the higher incidence of late major postoperative complications in the GA group might (partially) be the result of the higher incidence of early major postoperative complications: early major and late major postoperative complications in the GA group were 28.8% and 26.9%, while this was 13.6% and 16.0% in the NA group. This is substantiated by the fact that more than half of the late postoperative complications was permanent in the GA group (53.0%) while this was 30.4% in the NA group.

One final aspect to discuss is the fact that dedicated neuro-anesthesiologists more frequently used a combination of TIVA and volatile anesthetics than general anesthesiologists. The colleagues practicing this combination appreciate the stable anesthesia conditions. However, based on the (lack of) literature, we are unable to provide a good argument, why these differences in anesthesia technique may cause differences in outcome. While some authors support TIVA for brain tumor resection, others found recently no disadvantages for the use of volatile anesthetics in glioblastoma patients [21, 22]. The available evidence is not strong enough for a strict recommendation for one single anesthesia technique, and more recent insights suggest that an awake procedure might be even better [23].

### Limitations

The major limitation is the retrospective nature of this study. We expected a strong selection bias for patients operated under the supervision of a dedicated neuro-anesthesiologist. However, no significant differences in baseline characteristics were observed between groups, including well-known prognostic and predictive factors. Moreover, this study did only include GBM resections without mapping techniques, which might not be standard-of-care for some centers, especially for GBMs in or near eloquent areas. We therefore stress the importance of evaluating the benefit of the dedicated neuro-anesthesiologist in GBM resections with respect to (intraoperative) techniques for surgery in these areas.

To minimize most other possible bias correlated with the retrospective nature of this study, we underline the importance of a prognostic study to further investigate the potential of dedicated neuro-anesthesiologists in GBM surgery. We would like to invite our colleagues to cooperate in a prospective multicnter effort to validate the findings of this first proof-of-hypothesis study. In our opnion, such a study should focus on the etiology behind the perceived lower incidence of postoperative complications in GBM resections led by a neuro-anesthesiologist and the potential synergistic benefit of dedicated onco-neurosurgical teams. Moreover, the benefit of dedicated neuro-anesthesiology in combination with mapping techniques should be evaluated, ideally in subgroups of patients. The current study was conducted in a large high-volume university hospital with high-volume neurosurgeons (including experienced and specialized neuro-anesthesia and neuro-oncological teams, which in itself is beneficial for patient outcomes); nevertheless, large university hospitals with a comparable study setting, patient selection and local procedures can expect a robustness in the external validity of our findings.
